# HO-1 in Bone Biology: Potential Therapeutic Strategies for Osteoporosis

**DOI:** 10.3389/fcell.2021.791585

**Published:** 2021-11-30

**Authors:** Xueman Zhou, Wenxiu Yuan, Xin Xiong, Zhenzhen Zhang, Jiaqi Liu, Yingcheng Zheng, Jun Wang, Jin Liu

**Affiliations:** ^1^ State Key Laboratory of Oral Diseases and National Clinical Research Center for West China Hospital of Stomatology, Sichuan University, Chengdu, China; ^2^ Lab for Aging Research, State Key Laboratory of Biotherapy and National Clinical Research Center for Geriatrics, West China Hospital, Sichuan University, Chengdu, China

**Keywords:** bone remodeling, osteoporosis, pharmacological therapeutics, HO-1 inducer, heme oxygenase 1 (HO-1)

## Abstract

Osteoporosis is a prevalent bone disorder characterized by bone mass reduction and deterioration of bone microarchitecture leading to bone fragility and fracture risk. In recent decades, knowledge regarding the etiological mechanisms emphasizes that inflammation, oxidative stress and senescence of bone cells contribute to the development of osteoporosis. Studies have demonstrated that heme oxygenase 1 (HO-1), an inducible enzyme catalyzing heme degradation, exhibits anti-inflammatory, anti-oxidative stress and anti-apoptosis properties. Emerging evidence has revealed that HO-1 is critical in the maintenance of bone homeostasis, making HO-1 a potential target for osteoporosis treatment. In this Review, we aim to provide an introduction to current knowledge of HO-1 biology and its regulation, focusing specifically on its roles in bone homeostasis and osteoporosis. We also examine the potential of HO-1-based pharmacological therapeutics for osteoporosis and issues faced during clinical translation.

## Introduction

Osteoporosis, the most common bone disorder, is characterized by decreased bone mineral density (BMD), deterioration of bone microarchitecture and poor mechanical properties, resulting in increased vulnerability to fractures (NIH Consensus Development Panel on Osteoporosis Prevention and Therapy, 2000; van den Bergh et al., 2012; Vestergaard et al., 2007). Osteoporosis affects more than 200 million patients worldwide and its complications, especially osteoporotic fractures, can markedly reduce mobility and quality of life, increasing mortality, thus causing huge social and economic burdens (Blume and Curtis, 2011).

Pathologically, osteoporosis is the result of an imbalance in bone remodeling which is a dynamic process that involves both bone formation and resorption (Langdahl et al., 2016). For this reason, current pharmacological treatments of osteoporosis primarily are antiresorptive (inhibiting the osteoclasts, e.g., estrogen and bisphosphonates), bone forming (stimulating the osteoblasts, e.g., parathyroid hormone) or dual acting (e.g., romosozumab) (Langdahl, 2021). However, there is growing concern about the risk of adverse skeletal effects such as atypical femoral fractures and osteonecrosis of the jaw, as well as off-target effects in long-term use (Davis et al., 2016; Kim et al., 2016; Lv et al., 2020). Therefore, continuing efforts remain focused on filling the unmet need for safe and effective preventative and/or therapeutic strategies. In recent decades, extensive studies regarding the etiological mechanisms underpinning osteoporosis have emphasized that oxidative stress, inflammation and cellular senescence contribute to the progression of osteoporosis, indicating a new class of treatment strategies (Callaway and Jiang, 2015; Hendrickx et al., 2015; Farr and Khosla, 2019; Saxena et al., 2021).

Heme oxygenases (HO) are thes enzyme responsible for the degradation of heme into free iron, which is rapidly exported from cells *via* ferroportin 1 (FPN1) or sequestered into ferritin for storage; biliverdin, which is converted to bilirubin by biliverdin reductase; and carbon monoxide (CO) (Tenhunen et al., 1968; Kumar and Bandyopadhyay, 2005). Heme oxygenase-1 (HO-1), is an inducible form of HO that is highly expressed in tissues responsible for heme metabolism, including bone marrow (Gozzelino et al., 2010). Mounting studies have suggested that activated HO-1 is associated with the prevention of various diseases, including cancer, diabetes, cardiovascular diseases and osteoarthritis (Ayer et al., 2016; Chiang et al., 2018; Rochette et al., 2018; Alcaraz and Ferrándiz, 2020). These beneficial effects might be attributed to the anti-inflammatory, antioxidative, antiapoptotic and cell-cycle regulatory effects of HO-1 through its metabolites. Recently, it has been postulated that management of the expression and activity of HO-1 could represent provide a new idea for osteoporosis treatment (Che et al., 2021). With these points in mind, this review discusses the current knowledge of HO-1 biology, focusing specifically on its roles in bone homeostasis and osteoporosis. We also highlight the potential pharmacological interventions under investigation that could alleviate osteoporosis by targeting HO-1.

## Pathogenesis and Molecular Mechanisms of Osteoporosis

Traditionally, osteoporosis is classified into primary and secondary types (Marcus et al., 2013). Primary osteoporosis is further divided into two subtypes: type I (postmenopausal osteoporosis), which is caused primarily by estrogen deficiency due to menopause, and type II (senile osteoporosis), which is primarily caused by aging. Secondary osteoporosis refers to bone disorders secondary to other medical conditions (renal osteodystrophy, diabetes-related osteoporosis, etc.) or adverse results of therapeutic interventions (glucocorticoid-induced osteoporosis etc.). (Riggs et al., 2001; Feng and McDonald, 2011). Regardless of type, the disease can be characterized as a disorder of bone remodeling resulting from the imbalance between bone-forming osteoblasts and bone-resorbing osteoclasts, and there are common underlying molecular mechanisms.

### Inflammation

Accumulating studies have revealed that, to a certain extent, osteoporosis can be regarded as an inflammatory disease (Arron and Choi, 2000; Lorenzo, 2000). On the one hand, there is a close association between an increased risk of osteoporosis and inflammatory conditions, such as rheumatoid arthritis, ankylosing spondylitis, and inflammatory bowel disease (Haugeberg et al., 2004; Mitra et al., 2000; Moschen and R, 2005). On the other hand, during osteoporosis, inflammatory mediators such as pro-inflammatory cytokines act on the skeletal cells directly or indirectly to promote the development of osteoporosis (McLean, 2009). Besides, recent evidence suggests both innate and adaptive immunocytes contribute to osteoporosis (Saxena et al., 2021; Wu et al., 2021).

Clinical studies have revealed that menopause results in elevation of pro-inflammatory cytokines while in the case of old age, senescent cells secrete a wide range of inflammatory cytokines, such as IL-1, IL-6, IL-8, TNF-α and IFN-γ, which correlate with the progression of osteoporotic bone loss (Pacifici, 1998; De Cecco et al., 2019). It has been reported that human peripheral-blood monocytes (PBMCs) from osteoporosis patients have 29–67% higher secretion of IL-1β, IL-6, and TNF-α in whole blood culture compared with healthy control subjects (Pacifici et al., 1987). Zheng et al. (Zheng et al., 1997) found statistically significant negative correlations between PBMC secretion of IL-1β, IL-6, and TNF-α and lumbar spine BMD, while a study in healthy population similarly showed an association between reduced BMD and inflammatory markers in the circulation system, especially IL-6 (Heinrich et al., 2003). Animal models also supported the pathological role for inflammation in osteoporosis as both TNF and TNF receptor 1 deficient mice present resistance to ovariectomy-induced bone loss (Roggia et al., 2001; Iqbal et al., 2006).

In osteoporosis models, elevated pro-inflammatory cytokines, including IL-6, IL-1 and TNF-α can induce bone loss by regulating osteoclastic differentiation and activation both directly and indirectly (Hofbauer et al., 2000). Specifically, IL-6 promotes osteoclastogenesis by increasing RANKL production in osteocytes and osteoblasts (Liu et al., 2005). IL-6 also helps osteoclast precursors to transmigrate from the bone marrow to the blood leading to systemic bone loss by upregulating S1PR2 [Sphingosine-1-phosphate (S1P)] receptor (Tanaka et al., 2014). Besides, IL-6 hampers WNT/β-catenin pathway by enhansing its antagonists, Dickkopf-related protein 1 (DKK1) and sclerostin (SOST), which inhibit osteoblast differentiation (Ohori et al., 2019; Li S. et al., 2020). Moreover, IL-6 appears to mediate TNF-α and IL-1β induced bone resorption (Devlin et al., 1998). TNF-α and IL-1β, both of which are pro-inflammatory cytokines, also play pro-osteoclastogenic and anti-osteogenic roles, especially in post-menopausal osteoporosis (Du et al., 2018; Luo et al., 2018). On the one hand, they promote RANKL dependent osteoclastogenesis *via* activation of transcription factors NF-κB, AP-1 and PI3k/AKT pathway (Lee et al., 2017; Luo et al., 2018). TNF-α also triggers SOST expression, which induces RANKL expression in osteocytes and further boosts osteoclastogenesis, while IL-1β increases CCR7 to enhance osteoclast migration and activation (Kim et al., 2012; Lee et al., 2017). On the other hand, both TNF-α and IL-1β inhibit the proliferation, differentiation and activity of osteoblasts (Ruscitti et al., 2015; Du et al., 2018). Additionally, other inflammatory cytokines, such as IFN-γ and IL-7 indirectly promote bone loss by activating T cells and increasing the levels of IL-1 and TNF-α (Takayanagi et al., 2000; Baek et al., 2006; Tang et al., 2018). All these findings support the notion that inflammation contributes to the development of osteoporosis.

### Oxidative Stress

Oxidative stress (OS) is caused by the accumulation of free radicals mainly due to inflammation and mitochondrial dysfunction (Sies et al., 2017). A growing amount of evidence suggests that OS, which increases with aging or menopause, can adversely affect bone homeostasis by favoring a pro-resorptive environment, and it is often detected in the bone tissue of osteoporosis patients (Manolagas, 2010; Marie, 2014). Reactive oxygen species (ROS), especially hydrogen peroxide and superoxide ions, are thought to affect the bone environment mainly by two means: increasing osteoclastic activity and suppressing osteoblastic functions (Wauquier et al., 2009).

Primarily, ROS promote osteoclast formation and activity by stimulating RANKL-induced NF-kB and MAPK activation (An et al., 2019). Secondarily, they induce the excessive production of osteoclastogenic cytokines such as IL-1, IL-6, TNF-α and IL-7 (Hyeon et al., 2013). It has been reported that p66^shc^, a redox enzyme responsible for the reduction of O_2_ to H_2_O_2_, is a critical mediator of the stimulating effects of OS on the of activation NF-κB, cytokine production, and osteoclastogenesis (Almeida et al., 2007b). Further, OS also affects the function of osteoblasts. ROS trigger the activation of FOXOs, a subset of forkhead proteins contributing to cell cycle arrest, and suppresses the WNT/β-catenin pathway in MSCs, thus impairing osteogenic differentiation and increasing the expression and activity of peroxisome proliferator-activated receptor (PPAR) γ, which increases adipogenesis at the expense of osteogenesis (Almeida et al., 2007a; Takada et al., 2009). Apart from FOXO/WNT signaling, in murine primary bone marrow-derived and other MSC cell lines, OS also inhibits hedgehog signaling to suppress osteogenic differentiation (Kim et al., 2010). Further, increased OS in bone stimulates apoptosis of osteoblasts and osteoblast progenitors (Ambrogini et al., 2010). These facts convincingly demonstrate that OS advances the occurrence of osteoporosis.

### Cell Senescence

Cellular senescence is a cell fate that involves irreversible cell cycle arrest, profound chromatin changes, apoptosis resistance and senescence-associated secretory phenotype (SASP) (Hayflick, 1965; Acosta et al., 2013). SASP is characterized by an increase in protein synthesis and secretion, including pro-inflammatory cytokines and chemokines, which has deleterious paracrine effects and is regarded as an essential mechanism of many age-related diseases (Coppé et al., 2010). In the past few years, there has been growing evidence suggesting that cellular senescence plays a vital role in the pathogenesis of osteoporosis (Liu and Wan, 2019). Firstly, markers of senescence p21, p16^Ink4a^, and p53 have been identified not only in mice but in aged bones from human biopsies (Dimri et al., 1995; Hernandez-Segura et al., 2019). Senescent cells (SnCs), including MSCs, osteoprogenitors, osteoblasts, osteocytes and immunocytes accumulate in the bone or bone marrow of aged mice and human (Farr et al., 2016; Piemontese et al., 2017). These SnCs, especially senescent osteoblasts and osteocytes acquire SASP to stimulate RANKL production, leading to enhanced osteoclastogenesis and the development of osteoporosis (Chen et al., 2013; Akkaoui et al., 2021). Elimination of senescent cells can be achieved pharmacologically by long-term senolytic treatment, by clearance of p16-positive genetic cells in *INK-ATTAC* transgenic mice, or by blocking SASP production through the targeted inhibition of Janus kinase pathway; each of these strategies can increase bone mass and improve microarchitecture in aged osteoporotic mice (Farr et al., 2017; Chandra et al., 2020; Sharma et al., 2020). Furthermore, DNA damage-induced cell cycle arrest leads to functional decline of osteoblasts by decreasing proliferation, limiting osteogenic differentiation, and impairing cell function (Abdallah et al., 2006; Kim et al., 2017). These findings point to targeting senescence as a novel strategy to alleviate osteoporosis.

## HO-1 and its Regulation

**FIGURE 1:**
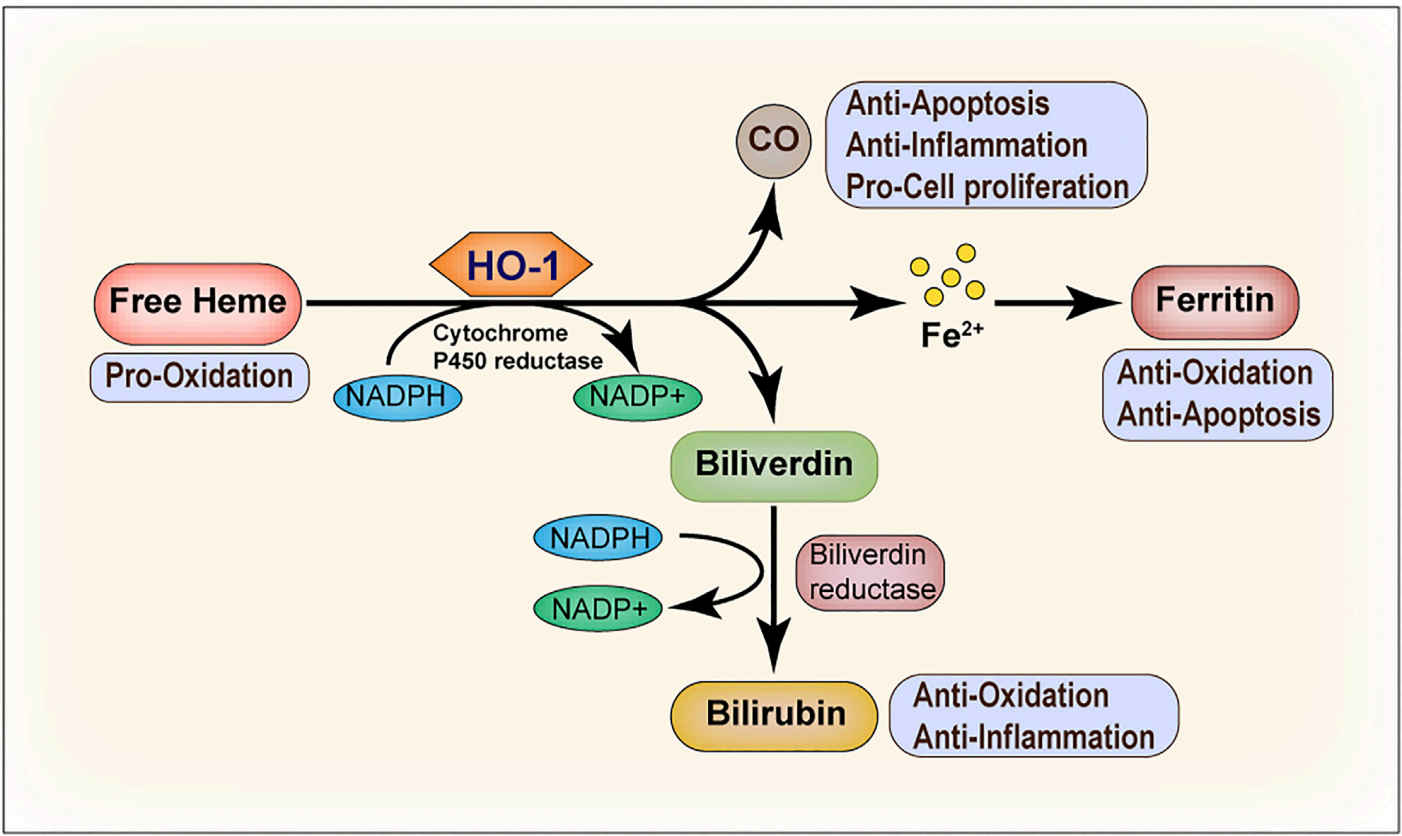
Heme oxygenase-1 (HO-1) catalyzes heme degradation. Free heme is degraded by HO-1, leading to the production of biliverdin, carbon monoxide (CO), and ferrous iron (Fe^2+^). Biliverdin is subsequently converted to bilirubin by biliverdin reductase, and Fe^2+^ is sequestered by ferritin. The degradation of heme and the conversion of biliverdin to bilirubin requires NADPH as the reducing agent. All the three end products, that is, biliverdin/bilirubin, CO, and Fe/ferritin, are cytoprotective. Under most conditions, biliverdin and bilirubin act as antioxidants. CO mainly inhibits the production of anti-inflammatory cytokines and upregulates the anti-apoptotic effectors. Ferritin serves as an antioxidant and suppresses cell apoptosis by binding and detoxifying ferrous iron.

HO-1, as a stress-inducible isozyme, catalyzes the degradation of heme into biliverdin (BV), carbon monoxide (CO), and free iron (Fe^2+^), releasing NADP^+^ and H_2_O (Maines, 1997). Under homeostatic conditions, HO-1 primarily maintains a low expression level or is absent from the body; however, it is highly upregulated in response to oxidative stress and provides protection against oxidative damage (Ryter and Choi, 2009). The induction of HO-1 exerts pleiotropic protective effects that are ascribed mainly to the biological activities of individual or cooperative effects of the metabolites (shown in Figure 1). Firstly, bilirubin (BR), derived by BV reduction, is a potent antioxidant able to scavenge ROS, thus preventing protein and lipid peroxidation. BR is also a key player in the control of inflammation as well as in the suppression of adaptive immunoreaction (McClung et al., 2021). Secondly, experimental evidence has established a firm basis for cytoprotective effects of CO involving the attenuation of inflammation, modulation of cell apoptosis and proliferation, as well as other cellular processes (Otterbein et al., 2016). Free iron, the third product of heme degradation induced by HO-1, reacts with hydrogen peroxide or lipid peroxides and produces numbers of reactive radicals, resulting in increased risk of many diseases and tissue injuries. However, it can be stored intracellularly by ferritin heavy chain (Van Lenten et al., 1995; Soares and Hamza, 2016). This means that ferritin serves as an antioxidant by binding and detoxifying ferrous iron.

**FIGURE 2:**
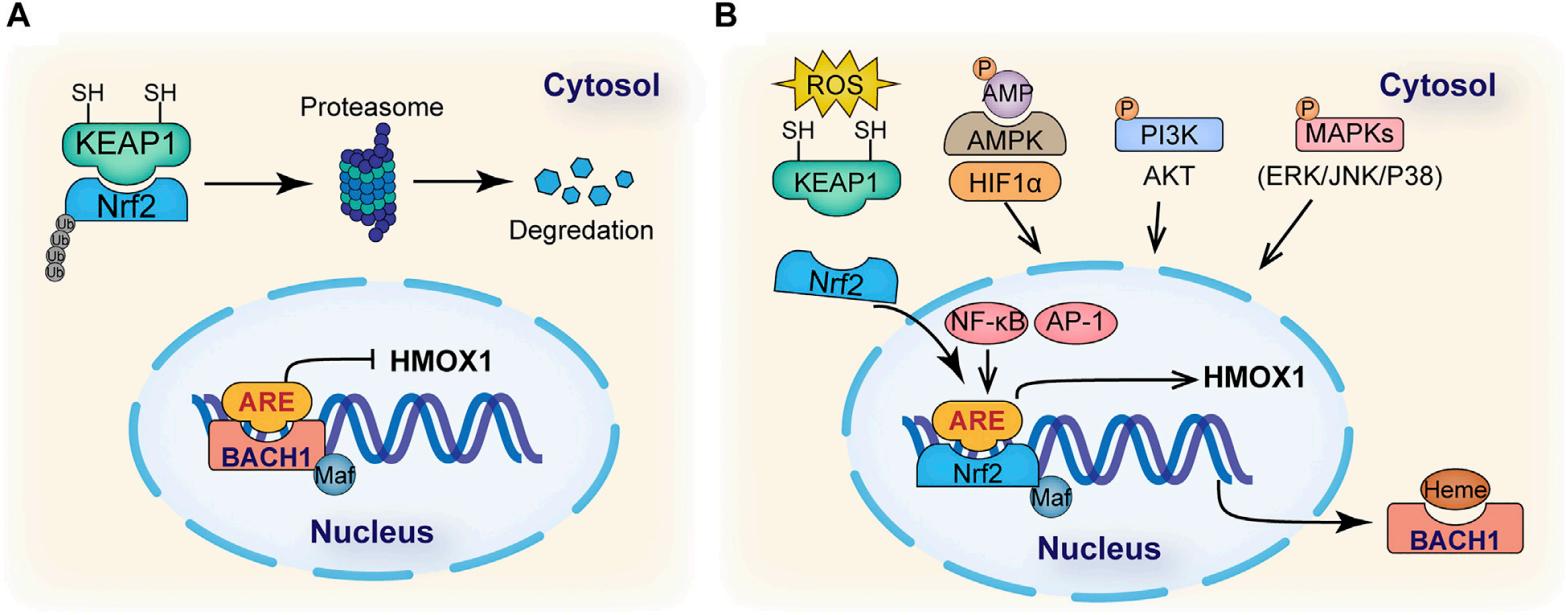
Mechanisms of HO-1 regulation. **(A)** Under basal conditions, nuclear factor erythroid 2- related factor 2 (NRF2) in cell cytosol binds to Kelch-like ECH- associated protein 1 (KEAP1), which promotes the ubiquitination and degradation of NRF2 in proteasomes. In the nucleus, BACH1 is bound to the ARE region in *HMOX1* gene promoter and represses its transcription. **(B)** Under stress, binding of heme molecules to BACH1 promotes its dissociation from the small Maf protein and ARE motif in the *HMOX1* gene promoter. ROS induces changes in KEAP1 cysteine residues, promoting the nucleus translocation of NRF2 to bind the ARE motif in *HMOX1* gene promoting its expression. Signaling cascades, such as AMP- activated protein kinase (AMPK), phosphatidylinositol 3-kinase (PI3K), mitogen-activated protein kinases (MAPKs), and transcription factors, such as hypoxia-inducible factor 1α (HIF1α), AP-1 and NF-κB, have also been reported to be involved in the regulation of HO-1 expression.

Given its essential role, understanding the regulatory mechanism of HO-1 expression has been the focus of considerable research. Current knowledge regarding the regulation of HO-1 activity depends heavily upon changes at the transcriptional level but also involves a range of post- or co-transcriptional regulated events (shown in Figure 2). The *HMOX1* gene which encodes HO-1 in mammals contains a motif known as Antioxidant Response Element (ARE) in its promoter site that can be recognized by a dimer composed of the BTB and CNC Homology 1 (Bach1) transcription factor together with Maf proteins. Under basal conditions, the Bach1-Maf dimer bounds to the ARE motif and represses the transcription of HO-1 while during OS, Bach1 is dislocated from ARE and exported from the nucleus to be degraded (Sun et al., 2002; Suzuki et al., 2004; Zenke-Kawasaki et al., 2007). Evidence coming from structure and sequence analysis of the promoter suggests that a group of redox-sensitive transcription factors activate HO-1, especially nuclear factor erythroid 2-related factor 2 (Nrf2) (Zhang et al., 2007). Under basal conditions, Nrf2 is sequestered in the cytoplasm by the kelch-like ECH-associated protein (Keap1) that hinders Nrf2 activity by facilitating the ubiquitylation and degradation of Nrf2 by the proteasome (Figure 2A) (Kim et al., 2013; Taguchi et al., 2011). However, in the presence of oxidants, ROS dissociate Keap1 from Nrf2 and translocate Nrf2 to the nucleus to bind AREs, promoting the expression of HO-1 (Korytina et al., 2019). Thus, the cellular induction of HO-1 is tightly regulated by extracellular conditions through this Nrf2/Keap1/Bach1 system. Other transcription factors are also known to bind ARE to stimulate HO-1 expression, such as activator protein-1 (AP-1), nuclear factor-kappa B (NF-κB) and hypoxia-inducible factor 1α (HIF1α) (Figure 2B) (Lavrovsky et al., 1994; Alam and Cook, 2007; Medina et al., 2020). Apart from the direct interaction with transcription factors, emerging evidence shows that microRNAs (miRs), the small noncoding RNAs involved in post-transcriptional modulation of gene expression, are also involved in the regulation of *HMOX1* gene expression either directly by decreasing the stability or translation of messenger RNA or indirectly by regulating the expression of upstream factors (e.g., Nrf2, Keap1, Bach1, etc.), which opens up a brand new horizon for the research of HO-1 regulation (Cheng et al., 2013; Kozakowska et al., 2014; Reziwan et al., 2019; Zhang et al., 2019).

It has been implied that many inducers regulate HO-1 expression *via* intermediate protein kinase pathways. For example, the mitogen-activated protein kinases (MAPKs), including extracellular signal-regulated kinase (ERK), JUN amino-terminal kinase (JNK) and p38 all act in regulating HO-1 expression (Martin et al., 2004; Mense and Zhang, 2006). The phosphatidylinositol 3- kinase (PI3K)-AKT pathway can also regulate HO-1 expression in response to oxidative stimuli and some alternative HO-1 inducers (Lin et al., 2007). Recently, AMP- activated protein kinase (AMPK) has been characterized as an HO-1 enhancer that interacts with Nrf2 when stimulated by many metabolic regulators and HO-1 inducers (Figure 2B) (Liu et al., 2011; Cho et al., 2018). In summary, induction of HO-1 can be achieved through various regulatory mechanisms under stress conditions.

### Biological Functions of HO-1 in Bone Remodeling

HO-1 is expressed in bone cells involved in the maintenance of bone homeostasis physiologically (Anselmino et al., 2020). A prior clinical study reported that total bilirubin, a metabolite of HO-1, positively correlated with serum calcium and BMD, and subjects with osteoporosis had a significantly lower total bilirubin level (Bian et al., 2013). Another study showed that in rheumatoid arthritis patients, serous bilirubin levels were much lower in those suffering bone damage than in those without bone loss (Peng et al., 2017). These clinical data suggest that HO-1 plays a key role in the maintenance of bone mass in humans.

Pioneering work on the role of HO-1 in bone biology was conducted in mice with global gene knockout (KO) of *Homx1*, *Nrf2* or *Bach1* (Florczyk-Soluch et al., 2018; Hama et al., 2012). Compared to wild-type controls, global *Homx1* KO mice revealed significantly decreased bone volume with a decline in the number of osteoblasts and osteogenic parameters. Also, plasma of *Homx1* ^−/−^ mice contained higher levels of C-telopeptide and tartrate-resistant acid phosphatase (TRAP) accompanied by an increase in active osteoclasts (Florczyk-Soluch et al., 2018; Ke et al., 2015; Zwerina et al., 2005). In the serum of *Nrf2* ^−/−^ mice, where HO-1 expression was inhibited, levels of RANKL significantly increased while osteocalcin decreased (Maicas et al., 2011). Besides, *Nrf2* genetic deficiency boosted RANKL-induced osteoclastogenesis of bone marrow macrophages leading to bone resorption (Ibáñez et al., 2014). In *Bach1* -KO mice, in which the expression of HO-1 was upregulated, the activity and mineralization of osteoblasts increased while osteoclastogenesis-induced bone resorption *in vivo* was repressed (Sudan et al., 2019). All these findings support that HO-1 functions in bone remodeling. Meanwhile, multiple studies have revealed that HO-1 deficient mice display increased inflammation accompanied by tissue iron accumulation and increased susceptibility to OS (Poss and Tonegawa, 1997a, b). Interestingly, the expression of HO-1 decreases with age. Induction of HO-1 in either MSCs or osteoblasts can reduce the features of senescence and restore the regenerative function of SnCs (Liu et al., 2017; Szade et al., 2020).

So far, there remains a lack of knowledge concerning the specific role of HO-1 in the bone tissues *in vivo* due to a lack of bone-specific conditional gene knockout animal models. To fill this research gap and to examine how HO-1 exerts its effect on bone metabolism through actions on bone cells, a battery of *in vitro* models with pharmacological treatment (such as hemin and cobalt protoporphyrin IX (CoPP)) or genetic modification to inhibit or overexpress HO-1 in osteogenic or osteoclast cell linages have been developed (Chae et al., 2006; Vanella et al., 2010; Nowak et al., 2018; Pan et al., 2018; Ma et al., 2020). Generally, the activation of HO-1 positively controls bone metabolism by maintaining an intracellular redox balance and cellular defenses to inflammation (Kensler et al., 2007; Rana et al., 2012). Under OS, which is induced by inflammatory cytokines such as TNF-α or by metabolic disorders such as diabetes-induced high glucose conditions, upregulation of HO-1 is required for maintaining mitochondrial homeostasis and protecting osteoblasts from apoptosis (Chae et al., 2006; Takanche et al., 2020; Zheng et al., 2021). Studies have shown that HO-1 induction in bone marrow mesenchymal stem cells (BMSCs) enhances the expression of osteogenic differentiation-related markers such as Runx2, bone morphogenetic protein-2 (Bmp2), osteocalcin (Bglap), and collagen 1A (Col1a), while also increasing the ratio of OPG/RANKL (Lin et al., 2010). Baebagallo et al. (Barbagallo et al., 2010) inhibited the expression of HO-1 in BMSCs by siRNA and detected an increase in the adipogenesis marker, PPARγ, whereas HO-1 overexpression promoted osteogenic differentiation and reduced the adipogenic differentiation. Meanwhile, the activity of senescence-associated β-galactosidase and the expression of the senescence markers were significantly decreased upon HO-1 induction, indicating that HO-1 levels could be strategically manipulated to protect osteoblast from senescence to restore cell function (Clérigues et al., 2012). These findings above all underline that HO-1 plays an indispensable, positive role in the process of bone formation.

As for the formation and activation of osteoclasts, HO-1 acts as a suppressor. When RANKL is combined with its receptor, the downstream signaling cascades such as NF-κB and MAPKs are activated, resulting in the sequential activation of nuclear factor of activated T cells cytoplasmic 1 (NFATc1) and c-Fos, known as master regulators of osteoclast differentiation and maturation (Boyle et al., 2003). It is worth noting that RANKL stimulation can upregulate Keap1 and induce separation of Nrf2 from the nucleus, resulting in reduction of downstream HO-1 (Kanzaki et al., 2013). The inhibition of HO-1 in bone marrow-derived macrophages (BMMs) consequently leads to impaired osteoclast differentiation (Sakai et al., 2012). Thus, HO-1 downregulation is a critical step of RANKL-induced osteoclastogenesis. Meanwhile, a series of studies have identified HO-1 as a negative regulator of osteoclast differentiation. *In vitro*, hemin-induced HO-1 upregulation downregulates the expression of cfms, RANK, TRAF-6, and c-fos mediated by MAPK inhibition, resulting in a compromised response of osteoclast to RANKL (Zwerina et al., 2005). HO-1 activation also decreases NF-κB translocation and preventes bone loss accompanied by a significant decrease in the ratio of RANKL/OPG (Kim et al., 2019). HO-1 also reduces intracellular ROS levels of the osteoclast precursors by suppressing expression of NOX1 and TRAF6 (Ke et al., 2015). Furthermore, metabolic products of HO-1, including CO and bilirubin can reduce RANKL-induced osteoclastogenesis *via* inhibiting the ROS/NF-κB pathway (Bak et al., 2017). *In vivo*, increasing HO-1 expression alleviates loss of bone mass in OVX osteoporotic mice (Xiao et al., 2020). Besides direct inhibition, HO-1 may exert immunomodulatory effects on the production of immune and inflammatory factors, and negatively regulate the differentiation or function of osteoclasts (Castejón et al., 2017; Alcaraz and Ferrándiz, 2020).

Growing evidence shows that iron plays an important role in the regulation of bone metabolism. Iron deficiency negatively affects collagen synthesis and vitamin D metabolism (Toxqui and Vaquero, 2015). However, iron overload, a state of excessive iron storage seen especially in patients with thalassemia, hemochromatosis, or sickle cell disease, is closely related to osteoporosis by promoting osteoclast differentiation and suppressing the proliferation and differentiation of osteoblasts (Jeney, 2017). In this regard, iron which is induced by HO-1-mediated heme degradation should be immediately seized by ferritin to avoid excess release and deposition. An *in vitro* study showed that HO-1 decreased the apoptotic rate of BMMSCs with iron overload through reducing intracellular ROS (Yu et al., 2018), indicating that HO-1 might participate in the regulation of iron metabolic homeostasis or attenuate iron-induced toxicity through a more comprehensive mechanism.

**FIGURE 3:**
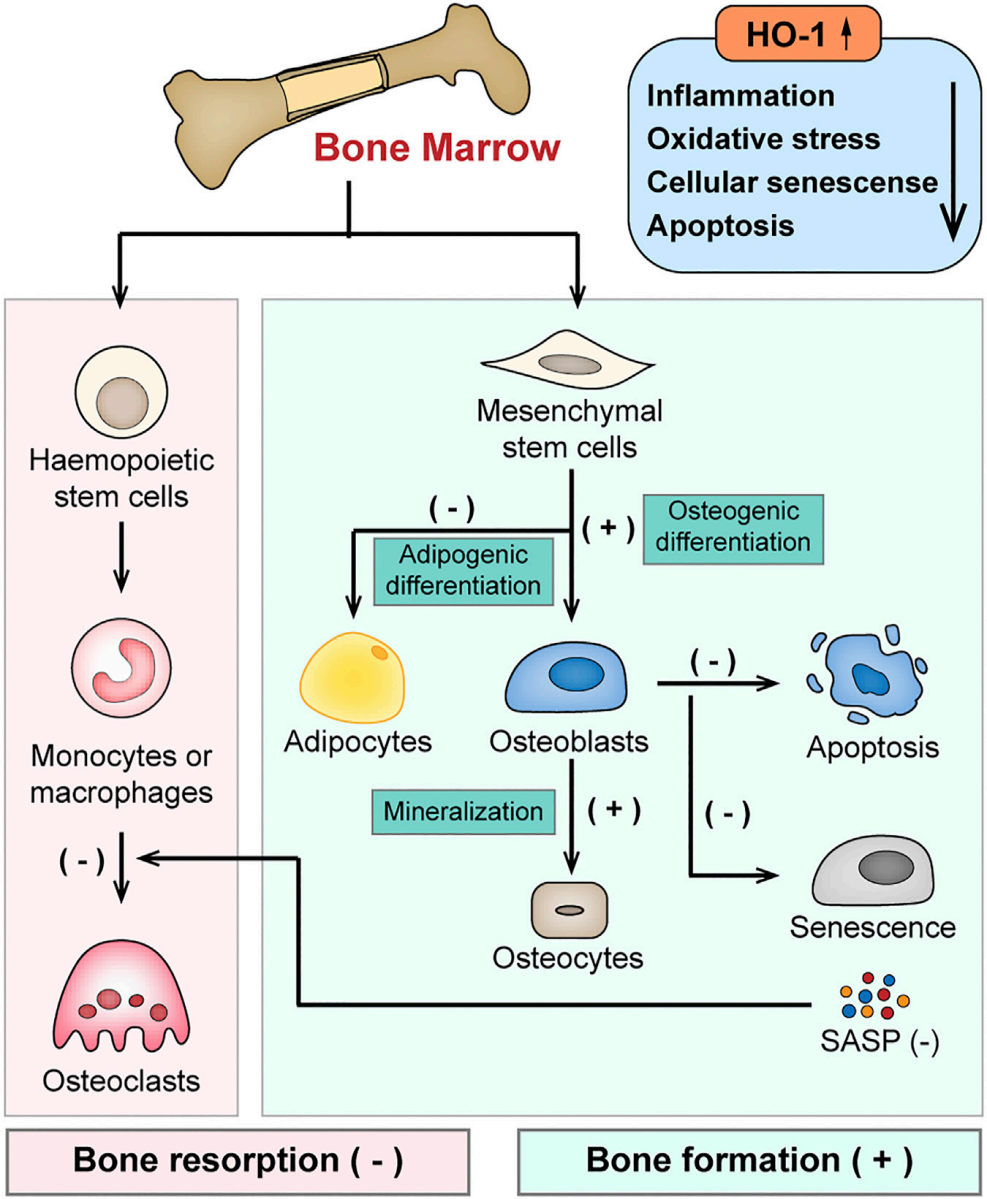
Schematic diagram of the protective effects of HO-1 in bone remodeling. HO-1 maintains a positive net balance of bone remodeling *via* dual-regulation of both osteoblasts and osteoclasts by alleviating inflammation, oxidative stress in the bone microenvironment. HO-1 regulates the differentiation of bone mesenchymal stem cells (BMSCs), enhances osteoblast function and inhibits the apoptosis and senescence of osteoblasts. It also negatively regulates the differentiation or function of osteoclasts.

In summary, by alleviating inflammation and oxidative stress, HO-1 provides a favorable remodeling microenvironment and maintains a positive net balance of bone *via* dual-regulation of both osteoblasts and osteoclasts (shown in Figure 3).

## Therapeutic Modulation of HO-1 in Osteoporosis

Despite the studies mentioned above supporting the notion that HO-1 is a novel therapeutic target for bone diseases, especially osteoporosis, it remains challenging for HO-1-based therapies to go into clinical application. Traditional HO-1 inducers such as metalloporphyrin are unsuitable for clinical use due to the significant toxicity (Suresh et al., 2003). Furthermore, clinical use of HO-1 metabolites, such as CO, is also hindered by concerns about toxicity and bioavailability (Bauer and Pannen, 2009). Thus, it is urgent to develop safer and more tolerable alternatives to HO-1 inducers and metabolites. In the following sections, we select some promising candidates and discuss their biological effects, as revealed by *in vitro* or *in vivo* studies (see Table 1 and Table 2).

**TABLE 1:** Pharmacological treatment towards HO-1 induction for osteoporosis *in vitro*.

Type of HO-1 inducer	Specific drug or chemical	Cell type	Effects on cells	Mechanisms	Ref
Phytochemicals	Chlorogenic acid	MC3T3-E1 cells	Prevented DXM-induced apoptosis	Promoted Nrf2/HO-1 **anti-oxidative** pathway by activating p21	Han et al. (2019)
	Costunolide	C3H10T 1/2 cells	Promoted osteogenic differentiation and matrix mineralization	Activated HO-1 **anti-oxidative** pathway by activating ATF4	Jeon et al. (2017)
	Curcumin	Rat MSCs	Promoted osteogenic differentiation and inhibited adipogenic differentiation	Activated HO-1	Gu et al. (2012)
			Prevented DXM-induced apoptosis	Activated the ERK pathway	Chen et al. (2016a)
			Promoted osteogenic differentiation and matrix mineralization	Activated Wnt signaling	Chen et al. (2016b)
		Mouse ASCs	Prevented H_2_O_2_-induced apoptosis	Activated HO-1 **anti-oxidative** pathway	Cremers et al. (2014)
		MC3T3-E1 cells	Promoted osteogenic differentiation and matrix mineralization	Promoted Nrf2/HO-1 **anti-oxidative** pathway	(Xin et al., 2015; Bukhari et al., 2019; Li et al., 2020b)
		Mouse BMMs	Suppressed osteoclast differentiation	**Suppressed ROS production**	Kim et al. (2011)
		RAW 264.7 cells	Suppressed osteoclast differentiation	**Suppressed ROS production**	Xin et al. (2015)
	Erxian decoction	MC3T3-E1 cells	Prevented apoptosis	**Suppressed inflammation** via Akt/Nrf2/HO-1 pathway	Wang et al. (2019)
	Forsythoside-β	Mouse BMMs	Suppressed osteoclast differentiation	**Suppressed ROS production** *via* Nrf2-mir-214-3p-Traf3 axis	Hong et al. (2021)
	Geniposide	MC3T3-E1 cells	Prevented apoptosis and increased osteogenic genes expression	Promoted Nrf2/HO-1 **anti-oxidative** pathway	He et al. (2019)
	Glabridin	MC3T3-E1 cells	Prevented methylglyoxal-mediated apoptosis	Promoted Nrf2/HO-1 **anti-oxidative** and **anti-inflammation** pathway	Choi et al. (2016)
	Gomisin A	MC3T3-E1 cells	Promoted osteogenic differentiation and mineralization	Promoted Nrf2/HO-1 **anti-oxidative** pathway	Takanche et al. (2020)
	Hesperetin	RAW 264.7 cells	Inhibited the differentiation and activity of osteoclasts	Promoted Nrf2/HO-1 **anti-oxidative** pathway	Liu et al. (2019)
	Magnolol	RAW 264.7 cells	Inhibited the differentiation and activity of osteoclasts	Promoted Nrf2/HO-1 **anti-oxidative** pathway, and inhibited MAPK and NF-κB signaling	Lu et al. (2015)
	Neobavaisoflavone	MC3T3-E1 cells	Prevented dexamethasone-induced apoptosis and promoted osteogenic differentiation	**Suppressed ROS production** *via* CRNDE-mediated Nrf2/HO-1 pathway	Zhu et al. (2021)
	Pristimerin	Mouse BMMs	Inhibited osteoclastogenesis	Promoted Nrf2/HO-1 **anti-oxidative** and **anti-inflammation** pathway, and inhibited MAPK and NF-κB signaling	Qi et al. (2020)
	Puerarin	RAW 264.7 cells	Suppressed the differentiation and activity of osteoclasts	Promoted Nrf2/HO-1 **anti-oxidative** pathway, and inhibited MAPK and NF-κB signaling	Xiao et al. (2020)
	Quercetin	Fetal rat calvarial osteoblasts	Enhanced antioxidant response	Activated HO-1 **anti-oxidative** pathway	Messer et al. (2016)
	Resveratrol	Mouse BMMs	Inhibited osteoclastogenesis	**Suppressed ROS production** *via* HO-1 pathway, and inhibited MAPK and NF-κB signaling	Kim et al. (2015)
	Schisandrin A	Mouse BMMs	Inhibited osteoclastogenesis	Promoted Nrf2 **anti-oxidative** pathway, and inhibited NF-κB signaling	Ni et al. (2020)
	THSG	MC3T3-E1 cells	Inhibited apoptosis and promoted osteogenic differentiation	Promoted HO-1 **anti-oxidative** pathway, and inhibited NF-κB signaling	Cheng et al. (2019)
	YS-51S	ROS 17/28 osteoblast cells	Alleviated NO-mediated cell death	Promoted HO-1 **anti-oxidative** and **anti-inflammation** pathway, and inhibited NF-κB signaling	Chaea et al. (2007)
	Z-Guggulsterone	MC3T3-E1 cells	Reversed DXM-induced cell death and osteogenic inhibition	Promoted Nrf2/HO-1 **anti-oxidative** pathway	Xu et al. (2019)
Existing drugs	5-ALA	RAW 264.7 cells	Inhibited osteoclastogenesis	Promoted Nrf2/HO-1 **anti-oxidative** pathway	Kanzaki et al. (2017)
	DMF	RAW 264.7 Cells	Suppressed the differentiation and activity of osteoclasts	Promoted Nrf2 **anti-oxidative** pathway	Yamaguchi et al. (2018)
	Melatonin	MC3T3-E1 cells	Improved osteogenic differentiation	**Inhibited oxidative stress** and ferroptosis through activating the Nrf2/HO-1 pathway	Ma et al. (2020)
	Simvastatin	MG-63 cells	Prevented H_2_O_2_-induced apoptosis. Increased ALP activity	Promoted HO-1 **anti-oxidative** and **anti-inflammation** pathway	Yin et al. (2012)
CO-based therapies	CORM-2	Rat ASCs	Prevented apoptosis	Activated HO-1	Cremers et al. (2014)
		RAW 264.7 cells	Inhibited the formation and activity of osteoclasts	**Inhibited oxidative stress** and NF-κB signaling via HO-1/CO pathway	Bak et al. (2017)
Novel inducers	Itaconate	Mouse BMMs	Suppressed the formation and activity of osteoclasts	Promoted Nrf2 **anti-oxidative** and **anti-inflammation** pathway, and inhibited NF-κB signaling	Sun et al. (2019)
	RTA-408	Mouse BMMs	Suppressed the formation and activity of osteoclasts	Promoted Nrf2 **anti-oxidative** and **anti-inflammation** pathway, and inhibited NF-κB signaling	Sun et al. (2020)

DXM, dexamethasone; ATF4, activating transcription factor 4; MSCs, mesenchymal stem cells; ASCs, adipocyte stem cells; CRNDE, colorectal neoplasia differentially expressed; BMMs; bone marrow-derived macrophages; ROS, reactive oxygen species; mir, micro-RNA; Traf3, TNF-receptor associated factor 3; MAPK, mitogen activated protein kinases; THSG, 2,3,5,4′-tetrahydroxystilbene-2-O-β-D-glycoside; YS-51S (S)-6,7-dihydroxy-1-(β-naphthylmethyl)-1,2,3,4-tetrahydroisoquinoline; 5-ALA, 5-aminolevulinic acid; DMF, dimethylformamide; CO, carbon monoxide; CORM, CO-releasing molecules; RTA-408, omaveloxolone.

**TABLE 2:** Pharmacological treatment towards HO-1 induction for osteoporosis *in vivo*.

Type of HO-1 inducer	Specific drug or chemical	Animal model	Effects	Ref
Phytochemicals	Curcumin	OVX rats	Attenuated bone loss, reduced osteoclasts numbers, and increased bone strength	French et al. (2008); Hussan et al. (2012)
		OVX mice	Attenuated bone loss; reduced osteoclasts numbers	Kim et al. (2011)
		GIO rats	Increased BMD, enhanced bone mechanical strength, and improved trabecular microstructure	Chen et al. (2016a); Chen et al. (2016b)
		T2DOP rats	Improved bone biomechanical properties and preserved bone microarchitecture	Liang et al. (2020)
		HLS-induced osteoporotic rats	Alleviated reduction of bone mineral density, and preserved bone structure and mechanical strength	Xin et al. (2015)
	Erxian decoction	OVX rats	Attenuated bone loss and decreased TNF-α levels in OVX rats	Wang et al. (2019)
	Forsythoside-β	LPS-induced osteoporotic mice	Attenuated bone loss; reduced osteoclasts numbers	Hong et al. (2021)
	Hesperetin	LPS-induced osteoporotic mice	Reduced bone loss, reduced osteoclasts numbers and decreased the RANKL/OPG ratio	Liu et al. (2019)
	Lutein	OVX rats	Inhibited inflammation and oxidative stress	Li et al. (2018)
	Pristimerin	OVX mice	Ameliorated bone loss and reduced serous inflammatory cytokines	Qi et al. (2020)
	Puerarin	OVX mice	Alleviated bone loss, reduced osteoclasts numbers and ROS within bone tissues	Xiao et al. (2020)
	Resveratrol	Periodontitis rats	Alleviated bone loss, reduced osteoclasts numbers and circulating ROS	Bhattarai et al. (2016)
	Schisandrin A	OVX mice	Alleviated bone loss, reduced osteoclasts numbers and ROS within bone tissues	Ni et al. (2020)
	TF3	OVX mice	Inhibited oxidative stress and osteoclastogenesis	Ai et al. (2020)
	Z-Guggulsterone	GIO rats	Increased bone mineral density. Ameliorated bone biomechanics and microstructure	Xu et al. (2019)
Existing drugs	5-ALA	LPS-induced osteoporotic mice	Alleviated bone loss, reduced osteoclasts numbers	Kanzaki et al. (2017)
	DMF	LPS-induced osteolytic mice	Alleviated bone loss	Yamaguchi et al. (2018)
	Melatonin	T2DOP mice	Increased bone mineral density and ameliorated bone microstructure	Ma et al. (2020)
CO-based therapies	CORM-3	OVX mice	Alleviated loss of bone mass and microstructure. Reduced osteoclasts. numbers	Ibanez et al. (2012)
Novel inducers	Itaconate	OVX mice	Alleviated bone loss and reduced osteoclasts numbers	Sun et al. (2019)
	RTA-408	OVX mice	Alleviated bone loss and reduced osteoclasts numbers	Sun et al. (2020)

OVX, ovariectomy; GIO, glucocorticoid-induced osteoporosis; BMD, bone mineral density; T2DOP, type 2 diabetic osteoporosis; HLS, hind-limb suspension; LPS, lipopolysaccharide; RANKL, receptor activator of nuclear factor-κB ligand; OPG, osteoprotegerin; ROS, reactive oxygen species; TF3, theaflavin-3, 3′-digallate; 5-ALA, 5-aminolevulinic acid; DMF, dimethylformamide; CO, carbon monoxide; CORM-3, CO-releasing molecules-3; RTA-408, omaveloxolone.

### Phytochemicals

Phytochemicals are secondary metabolites found in various plants and herbal substances which have been widely studied as antioxidant and anti-inflammatory agents (Islam et al., 2016; Pandey et al., 2018; Shin et al., 2020). Certain phytochemicals, employed alone or combined with other agents appear to be safe and effective disease-modifying drugs. Many phytochemicals have been reported to exert protective effects on bone cells and osteoporosis animal models through activation of HO-1 (Haines et al., 2012; Su et al., 2013). Among them, the most promising drug candidates are curcumin and resveratrol, both of which have been suggested to be effective in the treatment of osteoporosis in laboratory, translational and clinical studies (see Table 3) (Deng et al., 2021; Moschen and Shakibaei, 2013; Sharan et al., 2009). In a double-blind randomized controlled trial (RCT), postmenopausal osteoporotic women treated with the combination of curcumin and alendronate showed significant increases in total hip, lumbar spine and femoral neck BMDs accompanied by increased bone turnover markers (Khanizadeh et al., 2018). Similarly, a 24 months, two-period crossover clinical RCT was conducted recently to evaluate whether resveratrol supplementation could strengthen bones in postmenopausal women. The results showed that resveratrol (75 mg, twice daily) positively augmented BMD in the lumbar spine and femoral neck together with a 7.24% reduction in CTx (C-telopeptide of type I collagen, a marker of bone resorption). Further, the increased BMD in the femoral neck accounted for a reduction in the 10 years probability of hip fracture risk (Wong et al., 2020). Another RCT evaluating effects of resveratrol treatment on bone in obese men with metabolic syndrome revealed that high-dose resveratrol supplementation (Oral treatment with 1,000 mg daily) stimulated bone formation or mineralization (Ornstrup et al., 2014). However, several considerations should not be ignored. Firstly, despite their effectiveness, some of these compounds can modulate different signaling pathways and do not have selectivity for HO-1, which might lead to undesired side effects. For instance, many phytochemicals have low bioavailability, partially due to their poor stability and solubility in the digestive tract, which ultimately compromises their clinical use (Dos Santos et al., 2011; Mukkavilli et al., 2017; Wong et al., 2021). Secondly, these phytochemicals appear to be effective only at supraphysiological concentrations far exceeding those achievable through a daily diet. To overcome the shortcomings of phytochemicals, structural modification and catalyst compound-based approaches including novel delivery systems have been used (reviewed by Dei Cas, Ghidoni and McClements (Dei Cas and Ghidoni, 2019; McClements, 2020)). In addition, nanotechnology-based formulations have been shown to be useful as therapeutic agents for preventing and treating osteoporosis (Heo et al., 2014). Even so, it is necessary for much more attention to be paid to questions of bioavailability, route of administration and effective dosages before phytochemical HO-1 inducers go into clinical translation.

**TABLE 3:** Clinical trials of HO-1 inducers for osteoporosis.

Drug	Study population	Method and dose	Changes in observation index	Ref
Curcumin	Postmenopausal women	Oral treatment; 110 mg/dose/day for 12 months; together with alendronate (5 mg/day dose)	BAP and CTx levels decreased; osteocalcin level increased; Total body, total hip, lumbar spine and femoral neck BMD indexes increased	Khanizadeh et al. (2018)
Resveratrol	Postmenopausal women	Oral treatment; 75 mg/dose; twice daily for 2 years	BMD of in the lumbar spine and femoral neck increased; CTx reduced; T-score and the 10-years probability of major and hip fracture risk reduced	Wong et al. (2020)
	Middle-aged obese men with metabolic syndrome	Oral treatment; 1,000 mg/day for 16 weeks	BAP increased; Lumbar spine trabecular volumetric bone mineral density increased	Ornstrup et al. (2014)

BAP, bone-specific alkaline phosphatase; CTx, C-telopeptide of type I collagen; BMD, bone mineral density.

### Existing Drugs

Some existing drugs currently used for the treatment of inflammation have been revealed to activate NRF2 and/or upregulate HO-1. For example, 5-aminolevulinic acid (5- ALA) has been widely applied in photodynamic therapy for the treatment of skin diseases and some tumors (Shi et al., 2021). NRF2 activation and HO-1 upregulation by 5-ALA also present therapeutic potential for osteoporosis. *In vitro*, 5-ALA suppresses RANKL-mediated nuclear translocation of Bach1 and upregulates nuclear NRF2, inducing HO-1 expression in mouse primary peritoneal macrophages and in RAW264.7 cells, in turn increasing antioxidant activity. This results in decreased osteoclastogenesis and inhibited bone resorption (Kanzaki et al., 2017). Similarly, dimethyl fumarate (DMF) has been reported to trigger Nrf2 signaling to induce HO-1 and has been applied in clinical trials for skin diseases and neurodegenerative diseases (Foresti et al., 2013; Lehmann et al., 2007). In macrophages, DMF attenuates RANKL-induced intracellular ROS, inhibits RANKL-mediated osteoclastogenesis and suppresses osteoclast function, thus protecting bone from destruction (Yamaguchi et al., 2018). Melatonin significantly improves the osteogenic capacity of MC3T3-E1 cells by reducing the level of ferroptosis through *via* Nrf2/HO-1 pathway and augments bone mass in type 2 diabetic osteoporotic rats (Ma et al., 2020). Through enhancing HO-1, simvastatin, a potent hypolipidemic drug, ameliorated H_2_O_2_-induced intracellular OS and cell apoptosis while increasing alkaline phosphatase (ALP) activity in MG-63 human osteoblastic cells. In addition, simvastatin inhibits nitric oxide synthase (NOS) activity and iNOS expression under OS to protect against osteoporosis in aged and OVX rats (Yin et al., 2012). These drugs could be suitable candidates for osteoporosis treatment as HO-1 inducers considering their existing regulatory approval and safety data.

### CO-Releasing Molecules

CO-releasing molecules (CORMs) are spatially and temporally controlled CO releasers that can target specific tissues and present an alternative to CO gas inhalation. The biological effects of CORMs have also been observed in various animal studies and preclinical trials; they act *via* upregulation of HO-1 and can prevent inflammation and apoptosis (Alcaraz et al., 2008). When pre-treated with CORM-2, MSCs’ resistance to H_2_O_2_-mediated apoptosis is significantly increased (Cremers et al., 2014). It has been shown that, both in RAW264.7 cells and BMMs, CORM-2 treatment inhibits RANKL induced osteoclastogenesis and osteoclastic resorption activity by reducing RANKL-induced NFATc1 expression *via* inhibition of IKK-dependent NF-κB activation and ROS production (Bak et al., 2017). *In vivo*, administration of CORM-3 in OVX osteoporotic mice strongly induces HO-1 expression and shows a potent protection of BMD and bone mass as well as microarchitecture. The protective effects could be attributed to an anti-inflammatory effect, as indicated by lower levels of serous inflammatory cytokines, including TNF-α and IL-6 (Ibanez et al., 2012). However, heavy metal-based carrier presents toxicity concerns and there remains uncertainty concerning what constitutes a safe and effective dose, so further research is required before CORMs are eventually translated into clinical use.

### Novel Inducers

Recently, there has been increasing focus on identification of novel HO-1 inducers that may hold potential as anti-osteoporosis therapies. RTA-408, a novel NRF2 activator, has shown clinical therapeutic potential for dermatitis (Reisman et al., 2014), solid tumors (Creelan et al., 2017) and mitochondrial myopathies (A et al., 2016). A recent report revealed that RTA-408 effectively attenuates OVX-induced bone loss in mice by inhibiting STING-dependent NF-κB signaling and subsequent osteoclastogenesis (Sun et al., 2020). Itaconate, an endogenous metabolite, has been demonstrated to activate NRF2 to induce downstream HO-1 through alkylation of KEAP1, and controls inflammation control in both murine and human macrophages (Lampropoulou et al., 2016; Mills et al., 2018). Itaconate also ameliorates the severity of bone loss in a mouse model of OVX-induced osteoporosis and restrains ROS production, inflammatory responses and osteoclastogenesis *via* inhibition of the E3 ubiquitin ligase (Hrd1) to dislocate Nrf2 from ubiquitin (Sun et al., 2019), suggesting it could be a promising candidate for osteoporosis treatment in the future.

## Conclusion

This review reveals the important roles of stress-induced HO-1 activity in bone homeostasis and disorders, most notably osteoporosis. HO-1 can effectively restore the balance of bone remodeling through directly regulating the survival, differentiation, and function of bone cells, as well as by exerting anti-inflammatory, anti-oxidative and immunoregulatory effects to modulate the bone remodeling microenvironment. It is therefore a promising novel target for the development of anti-osteoporotic therapies. Up to now, some phytochemicals, existing drugs, and CORMs as well as novel Nrf2 inducers have been reported to prevent bone loss by upregulating HO-1 expression. In addition, the accumulated preclinical evidence and ongoing clinical trials have the lain the foundation for HO-1 inducers to be used as anti-osteoporotic drugs. However, despite significant progress, from a clinical perspective, the therapeutic potential of HO-1 is yet to be realized, and many questions about how to optimize the efficacy and minimize undesired effects of HO-1 inducers remain to be answered. Thus, further studies should be pursued to investigate novel or alternative HO-1 inducers, as well as to repurpose existing drugs as HO-1 stimulators.
